# A New Nomogram for Predicting 30-Day In-Hospital Mortality Rate of Acute Cholangitis Patients in the Intensive Care Unit

**DOI:** 10.1155/2023/9961438

**Published:** 2023-08-10

**Authors:** Li-Na Pan, Shen-Ao Pan, Guang-Liang Hong, Kun-Wei Chen

**Affiliations:** ^1^Department of Anesthesiology, The First Affiliated Hospital of Wenzhou Medical University, Wenzhou 325000, China; ^2^The Second Clinical Medical College, Wenzhou Medical University, Wenzhou 325035, China; ^3^Department of Emergency, The First Affiliated Hospital of Wenzhou Medical University, Wenzhou 325000, China

## Abstract

**Purpose:**

Acute cholangitis (AC) is a widespread acute inflammatory disease and the main cause of septic shock, which has a high death rate in hospitals. At present, the prediction models for short-term mortality of AC patients are still not ideal. We aimed at developing a new model that could forecast the short-term mortality rate of AC patients.

**Methods:**

Data were extracted from the Medical Information Mart for Intensive Care IV version 2.0 (MIMIC-IV v2.0). There were a total of 506 cases of AC patients that were included. Patients were given a 7 : 3 split between the training set and the validation set after being randomly assigned to one of the groups. Multivariate logistic regression was used to create an AC patient predictive nomogram for 30-day mortality. The overall efficacy of the model is evaluated using the area under the receiver operating characteristic curve (AUC), the calibration curve, the net reclassification improvement (NRI), the integrated discrimination improvement (IDI), and a decision curve analysis (DCA).

**Results:**

Out of 506 patients, 14.0% (71 patients) died. The training cohort had 354 patients, and the validation cohort had 152 patients. GCS, SPO_2_, albumin, AST/ALT, glucose, potassium, PTT, and peripheral vascular disease were the independent risk factors according to the multivariate analysis results. The newly established nomogram had better prediction performance than other common scoring systems (such as SOFA, OASIS, and SAPS II). For two cohorts, the calibration curve demonstrated coherence between the nomogram and the ideal observation (*P*  >  0.05). The clinical utility of the nomogram in both sets was revealed by decision curve analysis.

**Conclusion:**

The novel prognostic model was effective in forecasting the 30-day mortality rate for acute cholangitis patients.

## 1. Introduction

Acute cholangitis is an infectious disease characterized by bile tract obstruction and bile bacteria growth, which can cause acute infection of the bile duct and systemic inflammation [1]. The typical characteristics of acute cholangitis are fever, right upper abdominal pain, and jaundice. In severe cases, septic shocks and mental state changes may occur [2]. In addition to the obvious local symptoms, patients with AC also have a strong systemic inflammatory response. Sepsis and septic shock, both potentially fatal complications of AC, can appear suddenly [2]. Acute cholangitis mortality rates are quite variable. It has been reported that the 7-day mortality rate for severe patients can reach 10%, while the 30-day mortality rate can reach 30% [3]. In previous decades, the mortality rate for severe acute cholangitis was even higher than 50% [4]. With the advancement of early detection of severe acute cholangitis, the widespread use of endoscopic retrograde cholangiopancreatography (ERCP) technology, and more aggressive surgical treatment in recent years, the 30-day all-cause mortality rate of acute severe cholangitis has reduced dramatically but still remains close to 10% [5]. Therefore, enhancing the early diagnosis and timely treatment of patients with severe acute cholangitis may further improve their survival.

Once upon a time, Charcot's triad and Reynolds' pentad were considered to be the most important criteria for the clinical diagnosis of acute cholangitis. Nevertheless, the limitations of using these criteria were extremely clear. Charcot's triad has excellent specificity but low sensitivity [6]. It has been reported that Charcot's triad is present in 60–70% of AC patients, while Reynold's pentad is found in just 3–5% [2]. Since the 2007 Tokyo Guidelines (TGs) suggested the severity-based categorization method to tell the difference between mild and severe AC patients, it has become much easier to find severe AC patients early and start treatment right away [7]. Now, the Tokyo Guidelines have been updated to the 2018 version (TG18), which is widely used in clinical settings because it has good sensitivity and specificity [8]. However, TG18 uses the TG13 diagnostic and severity assessment criteria, and no new evidence is added to the existing TG13 criteria. As a result, many hospitals have adopted new the Sepsis-3 criteria, which may affect the diagnosis and management of AC patients [9]. At the same time, some researchers have found that TG18 is less than 70% sensitive and specific in AC patients with severe sepsis [10]. So, there is an urgent need for new clinical evidence to improve the rate of early diagnosis of patients with critical acute cholangitis.

In this study, 506 patients with acute cholangitis were selected from the MIMIC-IV database for this retrospective study to analyze the key factors that may affect the short-term mortality of patients with AC and to construct a nomogram for prognosis to improve the prediction of overall survival of patients with AC.

## 2. Methods

### 2.1. Sources of Data

MIMIC-IV, a publicly accessible, integrated, deidentified, single-center clinical database created by MIT, served as the source of all the data for this investigation. From 2008 through 2019, the MIMIC-IV database collected information on 315,640 hospitalized patients. Inpatient data were obtained from Beth Israel Deaconess Medical Center (BIDMC). To protect patient privacy, all patient identifiers have been removed from the database. Demographic data, laboratory results, medication and treatment information, and other health-related data are all included in the database. The MIT Institutional Review Board has granted database access to one author who has successfully completed an ethical training course (Certificate number: 43700334).

### 2.2. Case Inclusion Criteria

**Figure 1:**
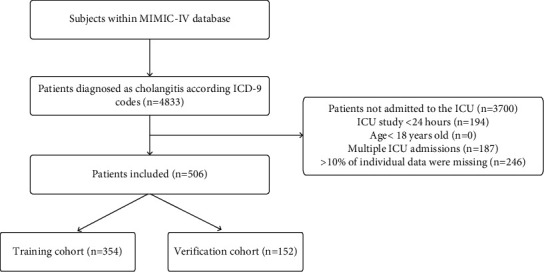
Flowchart of study cohort selection.

We used PostgreSQL, Navicat software, and SQL (structured query language) to extract data. Patients were identified with cholangitis by ICD-9 diagnostic codes (57451, 57491, 5761, and 5762). Patients were excluded from the trial if they met the following criteria: (1) were younger than 18 years old; (2) had a hospital stay of less than 24 hours; and (3) missing data rate >10%. If the patient has multiple ICU admissions, we only analyze the data from the first ICU admission. The flowchart of the inclusion and exclusion criteria is shown in Figure 1.

### 2.3. Data Collection

Basic vital signs, demographics, comorbidities, scoring systems, and laboratory test results were retrieved within 24 hours of patient admission to the ICU. Demographics include gender and age. Scoring systems include OASIS (oxford acute severity of illness score), GCS (Glasgow coma core), SAPS II (simplified acute physiology score II), and SOFA (sequential organ failure assessment). Basic vital signs included mean blood pressure, heart rate, SPO_2_, temperature, and respiratory rate. Laboratory tests included albumin, RDW (red blood cell distribution width), creatinine, AST/ALT (aspartate transaminase-alanine transaminase), AG (anion gap), bicarbonate, WBC (white blood cell count), BUN (blood urea nitrogen), blood potassium, blood glucose, blood sodium, hemoglobin, total bilirubin, blood calcium, platelet count, and PTT (partial thromboplastin time). Comorbidities include peripheral vascular disease, liver disease, diabetes, CHF (chronic heart failure), sepsis, chronic pulmonary disease, and malignant cancer.

### 2.4. Statistical Analysis

A 7 : 3 ratio of registered cases was randomly assigned to the training group and the test group. For variables that were not normally distributed, missing values were filled in with the median, whereas for variables that were normally distributed, missing values were filled in with the mean. The mean and standard deviation (mean ± SD) were normally distributed continuous variables, and the two groups were compared by the independent sample *T* test. We utilized the Mann–Whitney *U* test to compare continuous variables between the groups that did not have a normal distribution. These variables are provided as medians (quartiles) and are not normally distributed. We utilized the chi-square test or Fisher's exact test to compare categorical variables between the groups. Frequencies (%) are reported as categorical variables. 30-day mortality was the primary outcome of our study.

In the training cohort of patients with cholangitis, we utilized logistic regression to find variables that were independently linked with 30-day mortality. We included factors with a *P* value less than 0.1 in multivariate regression for further analysis and computed the estimated odds ratio (OR) and 95% confidence interval (CI). To find independent risk variables for the training cohort, we used the multiple logistic regression model and the backward stepwise regression technique. We used the variance inflation factor (VIF) to analyze multicollinearity between continuous variables in the final model, and we regarded collinearity as VIF's arithmetic square root ≥2.

Multivariate logistic regression was used to establish the new forecast model. We assessed the nomogram using the ROC curve and AUC value. To determine if the expected probability and the actual results agreed, we employed the calibration curve. A calibration curve that is almost diagonal is a good calibration curve.

In order to assess the nomogram's performance and contrast it with the previous model, we employed NRI (net reclassification improvement) and IDI (integrated discrimination improvement). The accuracy of both models was evaluated by NRI, while the IDI assessed the effectiveness of their improvement. The prediction model's clinical utility was further assessed using decision curve analysis (DCA). We used SPSS (22.0) and R to conduct statistical analysis and map data (4.2.1). Statistically significant was defined as *P*  <  0.05.

## 3. Results

### 3.1. Baseline Characteristics of Patients

The study included 506 eligible patients. Among 506 patients, 243 (48%) were female, 263 (52%) were male, and 71 (14%) died within 30 days. A 7−3 method was used to distinguish between the training group and the verification group. The results were as follows: there were 354 patients in the training group and 152 patients in the verification group. There was no significant difference in the characteristics of the two groups. There were 165 females (46.6%) and 189 males (53.4%) in the training group, with 50 deaths (14.1%) within 30 days. There were 78 females (51.3%) and 74 males (48.7%) in the verification group, with 21 deaths (13.8%) within 30 days. Baseline characteristics of patients with cholangitis are shown in Table 1.

### 3.2. Logistic Regression Analysis

We discovered that MBP, heart rate, GCS, breath rate, SPO2, albumin, BUN, AG, creatinine, platelet, bicarbonate, AST/ALT, blood glucose, WBC, blood calcium, blood potassium, PTT, liver disease, and peripheral vascular disease were risk factors in cholangitis patients by using univariate logistic regression analysis. By integrating these abovementioned variables into multivariate logistic analysis, we found that GCS, SPO2, albumin, AST/ALT, glucose, potassium, PTT, and peripheral vascular disease remained risk factors. The VIF is calculated among model variables, and the square root of the VIF among model variables is less than 2. This indicates that the model does not have multicollinearity.  Table 2 shows the results of the logistic regression analyses.

### 3.3. Prognostic Nomogram for 30-Day Mortality and the Nomogram's Performance

**Figure 2:**
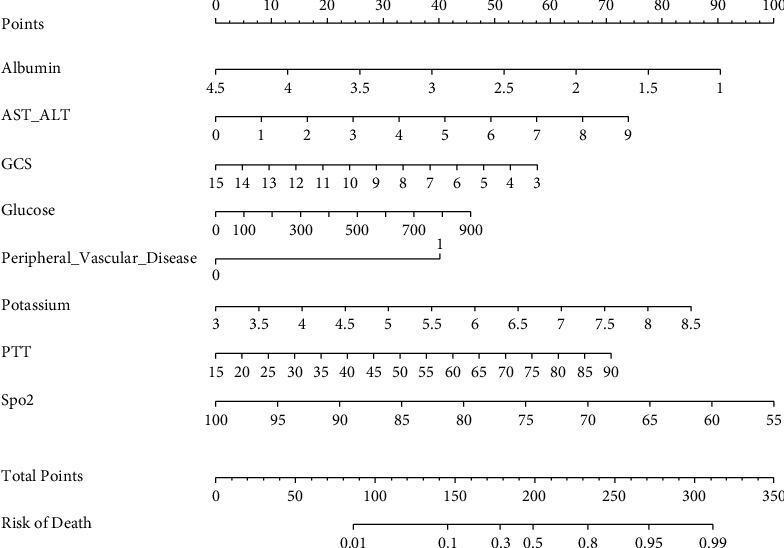
Nomogram predicting 30-day mortality in patients with cholangitis. To use a nomogram, draw a vertical line from each variable up to the point. The resulting value is the patient's score on that variable (i.e., “albumin = 1”=90 points). The scores for each variable were then summarized to obtain a total score corresponding to the 30-day probability of death predicted at the bottom of the nomogram. We then plotted a vertical line from the axis of the total point down to the 30-day survival probability, thus obtaining the 30-day survival probability for this patient. AST/ALT: aspartate aminotransferase-alanine aminotransferase; GCS: Glasgow coma scale; PTT: partial thromboplastin time.

**Figure 3:**
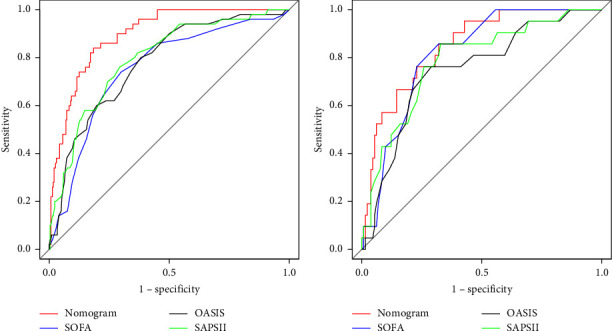
ROC curves for the nomogram, SOFA, OASIS, and SAPS II in the training cohort (a) and validation cohort (b). The nomogram includes albumin, AST/ALT, GCS, glucose, peripheral vascular disease, potassium, PTT, and SPO2. In both groups, the nomogram had higher AUC values than SOFA, OASIS, and SAPS II.

We used risk factors to build nomogram prediction models. The nomogram is shown in Figure 2. Analysis of the nomogram shows that the AUC of the training model is 0.896 (0.856∼0.935) (Figure 3(a)). The AUC of the training group was dramatically enhanced when compared to SOFA, OASIS, and SAPS II.  Table 3 summarizes the ROC curves of the nomogram and three prognostic scoring systems.

**Figure 4:**
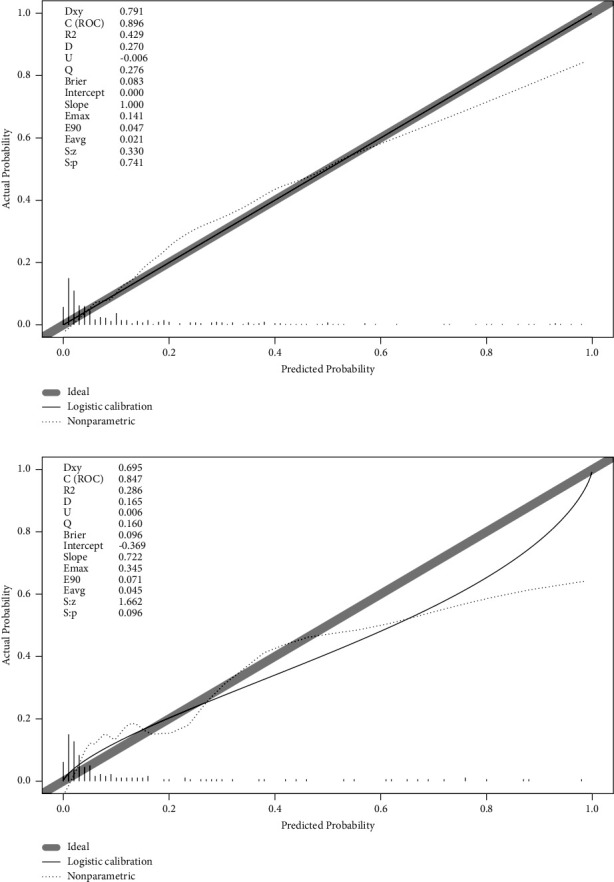
Calibration plots of the nomogram in the training cohort (a) and validation cohort (b). In both groups, although the apparent and corrected curves deviated slightly from the reference line, they also showed good agreement between observation and prediction.

We drew the calibration curve using the bootstrap method. If the calibration curve of the model is closer to the diagonal line, it indicates that the differentiation and prediction ability of the nomogram is good. The calibration curve of the training group is close to the diagonal line (Figure 4(a)). We verified the nomogram by inputting the data from the validation group. The AUC of the validation model is 0.847 (0.769–0.926) (Figure 3(b)). The calibration curve of the verification group is also close to the diagonal line (Figure 4(b)). This indicates that the model is effective in making predictions.

**Figure 5:**
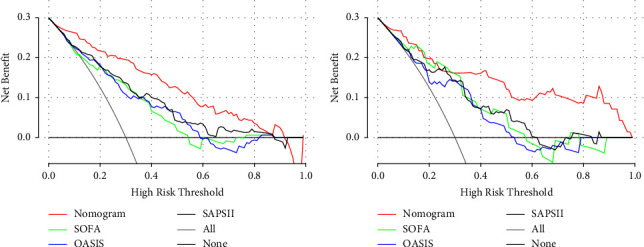
The decision curve analysis curves of medical intervention in patients with the nomogram, SOFA, OASIS, and SAPS II in the training cohort (a) and validation cohort (b). In both groups, the nomogram had superior standardized net benefit over SOFA, OASIS, and SAPS II.

Comparing the nomogram to OASIS, SOFA, and SAPS II revealed that the training group's NRI was 0.476, 0.353, and 0.216, respectively. The validation group's NRI was accordingly 0.507, 0.53, and 0.49. In addition, the training group's IDI was 0.212, 0.226, and 0.162, respectively. The validation group's IDI was 0.315, 0.265, and 0.258, respectively.  Table 3 summarizes the NRI and IDI of the training and validation groups. We found that both of the abovementioned values were positive numbers, which indicated that the model had strong discrimination. The nomogram's DCA curve is higher than the other three scoring systems when the threshold probability is between 0.4 and 0.8 (Figure 5). This suggests that the nomogram may guide clinical interventions with larger net benefits.

## 4. Discussion

Though the biliary tract is usually sterile, it can catch an infection if bacteria travel up from the digestive system or enter the body through the portal vein [11]. Normal hepatic biliary pressure is 12−15 cmH_2_O, slightly higher than normal extrahepatic bile duct pressure of 10−15 cmH_2_O, sustaining unidirectional bile flow from the common bile duct to the duodenum; these pressures are dynamically regulated by the Oddi sphincter [12]. However, this rule may be invalidated in the event of biliary blockage. The common bile duct stone is the most common cause of biliary obstruction [13]. When biliary blockage occurs, intrahepatic bile duct pressure will rapidly increase. If the pressure surpasses 30 cmH_2_O, liver biliary secretion will be considerably inhibited and intrahepatic pressure will increase, resulting in the destruction of the liver cell plate and the occurrence of cholangiovenous reflux [7]. Intestinal bacteria such as Escherichia coli, Klebsiella pneumoniae, and Enterococcus faecalis can retrogradely enter the systemic circulation via the common bile duct and trigger a systemic inflammatory response, resulting in severe septic shock and systemic multiorgan failure [14].

In this study, we established a new nomogram for predicting 30-day mortality in patients with AC based on a large population collected from the MIMIC-IV database. The variables included in the nomogram were GCS, SPO2, albumin, AST/ALT, glucose, potassium, PTT, and peripheral vascular disease. With its intuitive interface and improved accuracy, the nomogram can be widely used as a personalized risk prediction tool for clinicians and patients to calculate the AC risk according to their own conditions. So far, some AC prediction models have been proposed, but these models are still not comprehensive [15, 16]. Certain indicators, such as ventilator supporting time [15], biliary decompression treatment after admission [17], and bacterial culture results [16], were not accessible at the time of admission. There are also other indicators that are not routinely screened in emergency rooms, which impedes the evaluation of prediction models and severely limits the usage of physicians, such as lipocalin2 [18]. In our model, these indicators are easily accessible and have good practicability. In addition, the results of the AUC value, NRI, IDI, and DCA also prove that our model has a positive effect. In addition, this new nomogram's predictive performance is better than the current mainstream scoring system (SOFA, OASIS, and SAPS II) and was verified in our validation set.

TG18 is a clinical guideline that is often used to diagnose acute cholangitis and figure out how bad it is early on. It is important to note that in TG18, acute cholangitis that is accompanied by organ function impairment is considered severe. This includes cardiovascular, neurological, respiratory, renal, hepatic, and hematologic dysfunction. The severity assessment of acute cholangitis in TG18 also took into account factors such as fever, white blood cell count, age, bilirubin, and serum albumin [8]. In this study, we found new risk factors such as the AST/ALT ratio, PTT, blood glucose, blood potassium, and peripheral vascular disease. Hypoproteinemia, mental disorders, and hypoxemia remained consistent with TG18.

Fernando De was the first person to talk about the serum AST/ALT ratio as a sign of viral hepatitis in 1957 [19]. It has been demonstrated that the ratio of AST to ALT is a helpful prognostic indicator for individuals with severe acute viral hepatitis, alcoholic hepatitis, and cirrhosis [20, 21]. In recent years, various studies have demonstrated that the AST/ALT ratio is used to assess not only liver disease but also cardiovascular disease [22], the surgical prognosis of malignant tumors [23], and the occurrence of venous thromboembolism (VTE) [24]. According to the findings of our research, the relevance of the AST/ALT ratio is greater than that of bilirubin, which is somewhat different from TG18. In addition to the bias in the study samples, patients with underlying viral or alcoholic hepatitis may be a nonnegligible risk that accelerates the progression of infection.

Multiple studies have shown that in infectious diseases, even in the absence of diabetes, the mortality group has much higher blood sugar levels than the survival group [25, 26]. Infection can trigger a storm of inflammation that leads to insulin resistance and ultimately increases blood glucose levels [27]. Diabetes has also been reported to be strongly linked with the development of acute cholangitis in patients with common bile duct stones [28].

In sepsis, the large release of inflammatory factors activates the coagulation system, resulting in coagulation dysfunction and platelet depletion [29]. Coagulopathy is a prevalent sepsis-related complication that affects up to 80% of sepsis patients [30]. In this study, we discovered that PTT had a significant effect on short-term mortality in AC patients, and the predictive effect was superior to that of platelets, which differs from the recommended indicators by TG18. The possible reason is that the liver is an important organ that impacts the coagulation function, and infection mixed with liver function degradation affects the coagulation function more obviously than platelets. Moreover, liver disease was also an important risk factor in our univariate logistic regression analysis. Yet, we also noticed a decreased trend in platelets in the death group. Although platelets were not included in the prediction model, the effect of platelets on prediction can still be examined by increasing the sample size in future research.

In contrast to prior research, we first discovered that peripheral vascular disease (PVD) is an independent risk factor for AC patients. Data show that the risk of various cardiovascular events rises with the prevalence of PVD in the general population [31, 32]. PVD is an independent risk factor after percutaneous coronary intervention (PCI) and impacts long-term mortality in coronary artery bypass grafting patients [33, 34]. In addition to the fact that PVD patients are more likely to experience cardiovascular events while hospitalized, patients with peripheral vascular disease may take oral antiplatelet drugs for an extended period of time, thereby increasing their risk of bleeding and mortality.

Blood potassium disorder is one of the most common electrolyte disorders in clinic and is closely related to ICU patients' mortality [35, 36]. Abnormal potassium levels can lead to severe arrhythmia and myasthenia. According to studies, potassium channel blockade reduces organ damage and mortality caused by sepsis [37]. Meanwhile, people with abnormal blood potassium may also be connected with severe chronic disorders, such as renal failure and malignant tumors, thereby diminishing their ability to fight infection.

Our study provides a number of advantages. First, we discovered that a significant independent risk factor absent from the early AC model is peripheral vascular disease. Second, using simple-to-obtain parameters, we then build the nomogram above. This nomogram provides the probability of AC short-term mortality outcomes and enables practitioners to evaluate patient outcomes. Traditional disease severity scores are vastly inferior to our model's performance. This nomogram can help clinicians identify patients with severe cholangitis earlier and reduce the mortality of those patients through more aggressive treatment.

There are still limitations that must be considered. Initially, these data were obtained from a public database from 2008 to 2019. Therefore, the model needs to be externally validated using multicenter clinical data. Second, because some important indicators were missing by more than 20%, they were omitted from this study, resulting in the omission of some crucial clinical criteria. Third, our study did not account for the interaction or nonlinear relationship between covariates and outcomes; hence, the complexity of the relationship between covariates and outcomes was unknown. Finally, our prediction model is nondynamic, and it may be more reasonable to explore the relationship between time series variables and short-term in-hospital mortality in AC patients.

## 5. Conclusion

This study identified eight independent risk variables for the short-term mortality risk in AC patients and then used them to develop a nomogram. The results of this study can provide clinical reference for the early identification of patients with severe acute cholangitis.

## Figures and Tables

**Table 1:** Baseline characteristics of patients with cholangitis.

Variables	All (*n* = 506)	Training cohort (*n* = 354)	Validation cohort (*n* = 152)	*P* value
*Demographic*				
Age (year)	72.92 (60.86∼83.17)	72.52 (60.93∼82.73)	74.12 (59.89∼84.67)	0.477
Gender (*n*, %)				0.331
Female	243 (48%)	165 (46.6%)	78 (51.3%)	
Male	263 (52%)	189 (53.4%)	74 (48.7%)	
*Vital signs*				
Heart rate (beats/min)	71 (63∼84)	72.5 (63∼85)	70 (61∼80)	0.075
MBP (mmHg)	55 (49∼62)	55 (49∼62)	56 (49∼62)	0.539
Breath rate (breath/min)	27.75 (24∼32)	28 (24∼32)	27 (24∼31)	0.343
T (°C)	36.33 (35.89∼36.61)	36.36 (35.89∼36.61)	36.31 (36∼36.56)	0.729
SPO2 (%)	92.5 (90∼94)	93 (90∼95)	92 (90∼94)	0.197
*Prognostic scoring system*				
GCS	14 (12∼15)	14 (11∼15)	14 (13∼15)	0.622
SOFA	6.5 (4∼10)	7 (4∼10)	6 (4∼9)	0.448
OASIS	33 (27∼40)	33 (28∼41)	33 (27∼40)	0.511
SAPS II	43 (32.75∼52)	43 (33.75∼52)	42 (32∼51)	0.488
*Laboratory findings*				
Albumin (g/dL)	2.9 (2.4∼3.3)	2.85 (2.4∼3.2)	2.9 (2.5∼3.3)	0.642
BUN (mg/dL)	25 (16∼40)	26 (16∼42.25)	22 (16∼36.75)	0.125
RDW (%)	15.3 (14.1∼17.2)	15.3 (14.28∼17.23)	15.2 (13.9∼17)	0.103
AG (mEq/L)	16 (14∼19)	17 (14∼20)	16 (14∼19)	0.278
Bicarbonate (mEq/L)	21 (18∼23)	21 (17∼23.25)	21 (18∼23)	0.575
AST/ALT	1.27 (0.85∼1.75)	1.29 (0.87∼1.75)	1.23 (0.82∼1.73)	0.838
Creatinine (mg/dL)	1.15 (0.8∼1.8)	1.2 (0.8∼1.9)	1.1 (0.8∼1.7)	0.304
Glucose (mg/dL)	138 (111∼188.25)	139 (112∼189.25)	136 (109∼186)	0.7
Hemoglobin (g/dL)	9.87 ± 1.89	9.89 ± 1.86	9.81 ± 1.95	0.684
WBC (109/L)	10.7 (7∼15.83)	10.95 (6.98∼16)	10.25 (7∼15.5)	0.999
Platelet (109/L)	197 (132.75∼294)	194 (130∼288.25)	209 (139.25∼305.75)	0.248
Calcium (mmol/L)	7.7 (7.2∼8.2)	7.7 (7.2∼8.3)	7.7 (7.1∼8.2)	0.556
Sodium (mmol/L)	137 (134∼139)	137 (133∼139)	138 (135∼140)	0.068
Potassium (mmol/L)	3.6 (3.3∼4)	3.7 (3.4∼4)	3.6 (3.3∼3.9)	0.241
Bilirubin (mg/dL)	3.8 (2∼7.2)	3.85 (2∼7.2)	3.5 (1.8∼7.18)	0.447
PTT (s)	31 (27.5∼36.03)	31 (27.78∼36.1)	31 (27.33∼35.9)	0.56
*Comorbidities*				
CHF (*n*, %)	104 (20.6%)	72 (20.3%)	32 (21.1%)	0.855
Chronic pulmonary disease (*n*, %)	109 (21.5%)	77 (21.8%)	32 (21.1%)	0.861
Malignant cancer (*n*, %)	149 (29.4%)	103 (29.1%)	46 (30.2%)	0.792
Liver disease (*n*, %)	125 (24.7%)	93 (26.3%)	32 (21.1%)	0.212
Peripheral vascular disease (*n*, %)	37 (7.3%)	25 (7.1%)	12 (7.9%)	0.742
Diabetes (*n*, %)	168 (33.2%)	123 (34.7%)	45 (29.6%)	0.26
Sepsis (*n*, %)	431 (85.2%)	302 (85.3%)	129 (84.9%)	0.898

MBP: mean blood pressure; GCS: Glasgow coma scale; SOFA: sequential organ failure assessment; OASIS: oxford acute severity of illness score; SAPS II: simplified acute physiology score II; BUN: blood urea nitrogen; RDW: red blood cell distribution width; AG: anion gap; AST/ALT: aspartate aminotransferase-alanine aminotransferase; WBC: white blood cell; PTT: partial thromboplastin time; CHF: chronic heart failure.

**Table 2:** Univariate and multivariate logistic regression analysis in patients with cholangitis.

	Univariate analysis			Multivariate analysis		
	OR (95% CI)	B	*P* value	OR (95% CI)	B	*P* value
Age (year)	1.017 (0.995∼1.039)	0.017	0.131			
Gender (*n*, %)	1.243 (0.679∼2.276)	0.217	0.481			
GCS	0.792 (0.737∼0.85)	−0.234	<0.001	0.826 (0.756∼0.903)	−0.191	<0.001
*Vital signs*						
Heart rate (beats/min)	1.025 (1.006∼1.043)	0.024	0.009			
MBP (mmHg)	0.964 (0.942∼0.985)	−0.037	0.001			
Breath rate (breath/min)	1.065 (1.023∼1.109)	0.063	0.002			
T (°C)	0.895 (0.558∼1.434)	−0.111	0.644			
SPO2 (%)	0.916 (0.868∼0.967)	−0.088	0.001	0.933 (0.87∼1.002)	−0.069	0.055
*Laboratory findings*						
Albumin (g/dL)	0.284 (0.166∼0.485)	−1.26	<0.001	0.378 (0.198∼0.72)	−0.974	0.003
BUN (mg/dL)	1.013 (1.003∼1.022)	0.012	0.008			
RDW (%)	1.071 (0.951∼1.206)	0.069	0.257			
AG (mEq/L)	1.113 (1.051∼1.179)	0.108	<0.001			
Bicarbonate (mEq/L)	0.9 (0.847∼0.957)	−0.105	0.001			
AST/ALT	1.785 (1.347∼2.365)	0.579	<0.001	1.436 (1.017∼2.028)	0.362	0.040
Creatinine (mg/dL)	1.198 (1.015∼1.412)	0.18	0.032			
Glucose (mg/dL)	1.003 (1∼1.005)	0.003	0.057	1.004 (1∼1.007)	0.004	0.036
Hemoglobin (g/dL)	0.922 (0.783∼1.086)	−0.081	0.33			
WBC (10^9^/L)	1.034 (0.996∼1.074)	0.034	0.083			
Platelet (10^9^/L)	1.002 (1∼1.004)	0.002	0.081			
Calcium (mmol/L)	0.761 (0.563∼1.027)	−0.274	0.074			
Sodium (mmol/L)	0.971 (0.915∼1.03)	−0.029	0.334			
Potassium (mmol/L)	3.9 (2.236∼6.802)	1.361	<0.001	3.029 (1.543∼5.947)	1.108	0.001
Bilirubin (mg/dL)	1 (0.963∼1.039)	0.0002	0.992			
PTT (s)	1.056 (1.027∼1.087)	0.055	<0.001	1.042 (1.007∼1.079)	0.041	0.018
*Comorbidities*						
CHF (*n*, %)	1.459 (0.73∼2.917)	0.378	0.285			
Chronic pulmonary disease (*n*, %)	0.649 (0.291∼1.447)	−0.433	0.29			
Malignant cancer (*n*, %)	1.449 (0.772∼2.72)	0.371	0.248			
Liver disease (*n*, %)	1.717 (0.911∼3.236)	0.541	0.094			
Peripheral vascular disease (*n*, %)	2.587 (1.021∼6.556)	0.95	0.045	5.491 (1.771∼17.022)	1.703	0.003
Diabetes (*n*, %)	1.066 (0.571∼1.99)	0.064	0.841			
Sepsis (*n*, %)	1.067 (0.452∼2.52)	0.065	0.882			

MBP: mean blood pressure; GCS: Glasgow coma scale; BUN: blood urea nitrogen; RDW: red blood cell distribution width; AG: anion gap; AST/ALT: aspartate aminotransferase-alanine aminotransferase; WBC: white blood cell; PTT: partial thromboplastin time; CHF: chronic heart failure.

**Table 3:** Receive operating characteristics curve of the nomogram.

Predictive model	AUC	95% CI	*P*value	NRI	95% CI	*P*value	IDI	95% CI	*P*value
*Training cohort*									
Nomogram	0.896	0.856∼0.935	<0.01						
SOFA	0.753	0.681∼0.826	<0.01	0.353	0.124∼0.581	<0.01	0.226	0.141∼0.31	<0.01
OASIS	0.775	0.708∼0.842	<0.01	0.476	0.269∼0.683	<0.01	0.212	0.135∼0.289	<0.01
SAPS II	0.794	0.729∼0.86	<0.01	0.216	−0.001∼0.434	0.051	0.162	0.077∼0.247	<0.01
*Validation cohort*									
Nomogram	0.847	0.769∼0.926	<0.01						
SOFA	0.814	0.736∼0.893	<0.01	0.53	0.237∼0.822	<0.01	0.265	0.133∼0.397	<0.01
OASIS	0.748	0.636∼0.86	<0.01	0.507	0.187∼0.827	<0.01	0.315	0.172∼0.458	<0.01
SAPS II	0.785	0.679∼0.89	<0.01	0.49	0.168∼0.812	<0.01	0.258	0.119∼0.398	<0.01

SOFA: sequential organ failure assessment; OASIS: oxford acute severity of illness score; SAPS II: simplified acute physiology score II; NRI: net reclassification improvement; IDI: integrated discrimination improvement.

## Data Availability

The datasets are publicly available in the https://mimic.physionet.org/.
